# The Prevention of the Humped Back of Spinal Disease

**Published:** 1900-09-22

**Authors:** 


### THE PREVENTION OF THE HUMPED BACK OF SPINAL DISEASE.

The humped back of spinal disease is, of course, an opprobrium, and it is small wonder that the surgeon is anxious to efface it. But, if he had given proofs of such laudable efforts and anxiety at the beginning of his treatment, says Mr. Owen, he would probably have had no hump to deal with. This is a matter of much importance, serving as it does to illustrate the feeble treatment which sometimes results from lack of self-confidence in the practitioner. Mr. Owen says: "I have no hesitation in saying that, even at the present time, the treatment of spinal disease in its early stages is too often half-hearted, and sometimes actually blameworthy. It may be urged by way of excuse that, at the very beginning of spinal disease, the symptoms are so equivocal that the practitioner hesitates even to whisper his opinion lest the disappearance of the symptoms should suggest that after all he is an alarmist. He knew that the girl had symmetrical pains in her chest, belly, and legs; he knew that she got easily tired at play, or that she was inclined to loll and lie about when others were full of activity, and that, regardless of nursery manners, she persistently sat at meals with her elbows on the table. He suspected spinal disease; he even told the parents that the girl should be kept quiet. He may actually have gone so far as to sketch out a plan of treatment which was designed to secure a certain amount of rest, but he was slack in seeing that even this small measure was carried out. In short, he had not the courage of his opinion, and the case was allowed to drift. Oh! for the spirit of Lady Macbeth, who called out to her weak-kneed spouse and fellow-practitioner:

'Infirm of purpose! Give me the daggers!'

I am a great admirer of Lady Macbeth. . . . I am fully aware that her character was not faultless . . . . but how splendid she would have been in the treatment of early spinal disease! There would have been no half-measures with her!" And the treatment required at this the very earliest stage is rest, absolute rest. To secure this in the case of a child there is only one way, and that is by making him lie flat in bed. Whatever may be done later on, the treatment must at any rate be inaugurated by the patient being imprisoned in a pillowless bed, and the medical man must not merely recommend but must carefully consider all the details, and make it his business to see that they are loyally and faithfully carried out. This is strong teaching, but it is undoubtedly correct. In every direction it is becoming manifest that early diagnosis is the key to success. But when the diagnosis is made the surgeon must have the courage of his opinion, and we are afraid that he must be prepared in many cases, even when his success is greatest, to hear it said that if he had but let things alone they would have come all right. The reward of success does not always take the form of gratitude.

